# Telephone messages for medication adherence and self-care for individuals with coronary heart disease

**DOI:** 10.1590/0034-7167-2024-0351

**Published:** 2025-11-03

**Authors:** Luize Maximo e Melo, Vinícius Batista Santos, Juliana de Lima Lopes

**Affiliations:** I Universidade de São Paulo. São Paulo, São Paulo, Brazil; II Universidade Federal de São Paulo. São Paulo, São Paulo, Brazil

**Keywords:** Coronary Disease, Medication Adherence, Health Education, Cell Phone, Validation studies, Enfermedad Coronaria, Cumplimiento de la Medicación, Educación en Salud, Teléfono Celular, Estudio de Validación

## Abstract

**Objective:**

to develop and analyze evidence of validity of telephone messages to promote medication adherence and self-care among patients with coronary artery disease.

**Method:**

a methodological study, conducted in three stages: I) literature review and development of messages; II) assessment of content evidence of validity by judges; III) assessment of evidence of validity based on response processes by patients undergoing percutaneous coronary intervention. The Content Validity Ratio was calculated to analyze evidence of validity.

**Results:**

twenty-nine telephone messages related to barriers to medication adherence and self-care were developed, which were assessed by 23 judges and 31 patients, observing adequate values of the Content Validity Ratio.

**Conclusion:**

the messages were developed and achieved adequate evidence of validity, and may be an educational tool for this population.

## RESUMEN

**Objetivo:**

desarrollar y analizar evidencia de la validez de los mensajes telefónicos para promover la adherencia a la medicación y el autocuidado entre pacientes con enfermedad arterial coronaria.

**Método:**

estudio metodológico, realizado en tres etapas: I) revisión de literatura y desarrollo de mensajes; II) valoración de la validez de contenido de la prueba por parte de los jueces; III) evaluación de la evidencia de validez basada en los procesos de respuesta de los pacientes sometidos a intervención coronaria percutánea. Se calculó el índice de validez de contenido para analizar la evidencia de validez.

**Resultados:**

se elaboraron 29 mensajes telefónicos relacionados con barreras a la adherencia a la medicación y al autocuidado, los cuales fueron evaluados por 23 jueces y 31 pacientes, observándose valores adecuados del Índice de Validez de Contenido.

**Conclusión:**

los mensajes fueron desarrollados y lograron adecuada evidencia de validez, pudiendo ser una herramienta educativa para esta población.

## INTRODUCTION

Cardiovascular diseases (CVDs) are the main causes of death in Brazil and worldwide. Among them, coronary artery disease (CAD) is responsible for a high rate of hospitalization^(1,2)^. To control and reduce morbidity and mortality rates due to CAD, it is necessary to implement preventive measures that encourage changes in lifestyle and patient adherence to therapeutic regimen, since non-adherence to treatment is associated with worse outcomes^(3)^.

Historically, treatment adherence is a complex concept that has migrated from authoritarian approaches to the acceptance of self-management of treatment by individuals^(4-8)^. Adherence is then defined as behavior that coincides with care plan agreed between healthcare professionals and patient^(5-8)^, with pharmacological adherence being defined by the use of at least 80% of prescribed medications, observing the schedule, dose and treatment time^(5-8)^.

Non-adherence to treatment is a highly complex issue, being related to aspects of different natures, i.e., linked to the treatment itself (complexity of pharmacotherapy, treatment duration and intensity of adverse reactions), to the health condition (chronic diseases and asymptomatic conditions), to the patient (cognitive and functional limitations, knowledge about health conditions and medications, physical and motor difficulties), to social and economic factors (lack of family and social support, cultural beliefs, lack of access to healthcare services, lack of access to medications), and to factors related to the system and the link with health team^(9)^.

Analyzing the behavior of prescription drug use requires broad approaches that consider the importance of self-care, self-management and self-monitoring in health^(4,7,8)^. Therefore, this research used Dorothéa Orem’s medium-range theory as a theoretical framework, which considers that self-care includes actions initiated and executed by individuals for their own benefit to maintain life and well-being, contributing to the purpose of integrity of functions and human development^(10-13)^. This theory describes that individuals want and can become capable of self-care, and nursing professionals can take on this task when they are not in a position to do so^(12)^ and act through health education, enabling adherence to preventive and therapeutic behaviors (health self-management) and monitoring of their health (self-monitoring), making them active agents of their own care and thus facilitating self-care^(10-13)^.

With the increase in technology and the popularization of mobile phone messaging resources (WhatsApp^®^) and legal regulations^(14-17)^, health monitoring via mobile phone services has been promoted, with the aim of promoting greater accessibility to users of healthcare services by providing a low-cost means of open and immediate communication, expanding access to healthcare, reducing costs and geographical barriers between patient and healthcare professional, and overcoming difficulties with treatment^(16-18)^.

In the literature, there are primary studies and systematic literature reviews that aimed to assess the effect of mobile phone messages, identifying that these interventions provide an increase in behavioral changes in relation to lifestyle (health self-care), increased medication adherence (health self-management) and better monitoring of clinical signs (health self-monitoring)^(19-25)^. In a recent review carried out by Cochrane (2024)^(26)^ on the use of telephone messages in health education for coronary artery disease patients, an important gap in studies demonstrating the effect of this intervention was highlighted, especially in low-income populations, and also the lack of studies assessing evidence of validity of these educational materials aiming at achieving positive health results^(26)^.

Given the fact that the insertion of information and communication technologies in educational contexts in health can be a facilitator for self-management, self-monitoring and self-care in health, it is necessary that these technologies be subjected to psychometric techniques in order to assess evidence of validity of these materials^(27,28)^. Therefore, to the best of the authors’ knowledge, no Brazilian study was identified that developed and analyzed evidence of validity of telephone messages aimed at medication adherence for patients with coronary disease with a focus on the main barriers related to adherence.

The pioneering nature of Brazilian nursing’s study is considered, both for linking a digital technological tool and for its methodological improvement and systematization, through the analysis of evidence of validity by both specialists and the target population.

## OBJECTIVE

To develop and analyze evidence of validity of telephone messages to promote medication adherence and self-care for patients with CAD.

## METHODS

### Ethical aspects

This research was conducted in accordance with national and international ethical guidelines and was approved by the *Universidade Federal de São Paulo* Research Ethics Committee. It was conducted ethically, confidentially and only with the formal authorization of each research participant, through the signing of an Informed Consent Form (ICF), obtained in writing and electronically.

### Study design

This is a methodological study whose systematic strategy uses existing knowledge of a phenomenon to elaborate, adapt, develop, improve and assess an instrument in terms of its reliability, validity, usefulness and replicability^(27,29,30)^. The literature highlights some stages for developing educational instruments/tools that start with the construction of evidence of content validity (conceptual structure/latent variable establishment, objective definition, tool item/topic construction, item/topic selection and organization, instrument/tool structuring, and assessment by a heterogeneous committee of judges), followed by the target population’s involvement to determine evidence of validity based on the response process^(27-30)^.

This study was carried out from May 2020 to January 2022. Data were collected from patients admitted to the Clinical Cardiology Units of a teaching hospital of the tertiary public healthcare service in the city of São Paulo.

### Population and sample

For the process of analyzing evidence of content validity, a committee of judges with a minimum specialist qualification and more than four years of experience in the specific area of cardiology or public health was selected^(27,29,30)^. Recruitment was carried out using a snowball method, with a first stratum based on knowledge of the authors of this research and selection on the *Lattes* Platform, in order to aggregate a heterogeneous contingent of specialists, taking place between May and November 2020.

In the phase of assessing evidence of validity based on response processes, patients were selected and monitored between March 2021 and January 2022. Patients hospitalized for some manifestation of CAD, aged 18 years or older, who underwent percutaneous coronary intervention for the first time, users of the mobile phone messaging system (WhatsApp^®^) and being able to speak and understand Portuguese were included. Patients who presented severe cognitive decline and/or previous psychiatric conditions reported in medical records were not considered for this stage. Patients who failed to receive messages or requested to leave the study, failed telephone contact to administer the questionnaires and interview at the end, and readmission due to a new ischemic event and death were considered lost to follow-up.

### Study protocol

The stages developed in this research were: 1) narrative review of literature and development of messages to be sent to mobile phones; 2) assessment of evidence of content validity by a committee of judges specialized in cardiology; 3) assessment of evidence of validity based on response processes by patients undergoing percutaneous coronary intervention.

Assessment of evidence of content validity is understood as the relationship between the material/instrument content developed with the phenomenon of interest, i.e., it is a verification of whether the material/instrument is intended for the main construct in relation to theoretical relevance, practical pertinence and clarity, in addition to general appearance assessment and even visual resources used in preparation^(29,30)^. Analysis of evidence of validity based on response processes is related to respondents’ perception of the material developed, and can be carried out, among other techniques, through interviews with the target population. This analysis is very important, as it is the first moment of contact between the tool and the target audience, seeking a balance between the perspective of judges and the population group. To this end, it is based on several types of indicators, such as comprehensibility, scope of material, general organization and relevance^(30)^.

#### First stage

The first stage of this study involved a narrative review of literature and preparation of messages. The review was conducted in the PubMed, SciELO and Virtual Health Library databases, using the subject descriptors “Coronary Disease”, “Medication Adherence” and “Health Education”. Articles on the subject developed in the last five years and deposited in these databases were selected. Article search and selection were identified by screening titles and abstracts performed by one of the authors of the study, who extracted educational interventions aimed at solving potentially modifiable adherence barriers and promoting self-care in the population of patients with CAD.

After identifying the educational interventions, the authors developed telephone messages containing text and figures. The figures were later finalized and drawn by a professional illustrator.

#### Second stage

Judges were invited by email, and the research objectives were explained. Upon acceptance, a Google Forms^®^ link was sent by email, which contained the electronic ICF, the sociodemographic and professional characterization questionnaire (age, sex, academic level and time of experience in care, teaching and/or research), followed by the instrument with the developed messages.

To analyze the messages, a four-point Likert scale was used, with a score of 1 being not adequate, 2 being moderately adequate, 3 being adequate, and 4 being totally adequate. Judges assessed each message in relation to theoretical relevance of the information contained in the messages, clarity of information, practical relevance, adequacy of figures with the text of messages, font size and sharpness^(27,28)^. At the end, a field was left for suggestions for changes and justifications for the score given.

#### Third stage

Patients were included on the day before hospital discharge. During this contact, the study objectives were explained, the ICF was read, delivered and signed. If accepted, the first instrument containing sociodemographic and clinical data was applied through a structured interview. This instrument contained data related to sex, age, level of education, marital status, weight and height, personal history, lifestyle habits, access to healthcare services, and data related to medication use (accountability for medication use and family support relationships). Subsequently, patients were asked to provide their mobile phone number for use on WhatsApp^®^. As soon as they were added to the study, patients received an initial message confirming the purpose of the study to verify that it had been sent correctly.

Educational messages were sent on the day after participants were discharged and continued for 94 days. In the first week after discharge, messages were sent daily; then, they were spaced out at three-day intervals. The resource of “joker messages” was also used, which were reinforcement and/or motivational content regarding changes in behavior and were repeated throughout the study in an interleaved manner. The 94-day period was established after the content evidence validity stage was completed and, then, the total number of messages was ready, which, interspersed with the joker messages and sent at the pre-defined interval, totaled 94 days of monitoring.

After this period, a telephone contact was made for an interview to assess the messages in relation to font size and type, adequacy of figures with the text, sharpness of images and understanding, usefulness, and adequacy and clarity of these messages. To this end, an instrument developed by the authors was applied containing a 4-point Likert-type scale, with score 1 being not adequate, 2, moderately adequate, 3, adequate, and 4, totally adequate. At the same time, a qualitative analysis of interview responses was performed through an open-ended question regarding the resource usability.

### Analysis of results, and statistics

The collected data were processed in a Microsoft Excel^®^ spreadsheet and analyzed by the researchers using the Statistical Package for the Social Sciences^®^ version 22.0. Descriptive statistics were performed on the variables presented, with categorical variables presented in absolute and relative frequencies, and numerical variables in mean and standard deviation or median and minimum-maximum values, according to the sample distribution by the Shapiro-Wilk normality test.

To analyze evidence of content validity in response processes, the Content Validity Ratio (CVR) was calculated for each assessed message indicator, according to the formula CVR = Ne- (N/2)/N/2, where Ne is the number of judges and patients who scored 3 and 4, and N is the number of respondents^(31)^.

The acceptable critical value was dependent on the number of responding judges, as recommended by Ayre in 2014, with a minimum number of five judges being recommended^(31)^. In the first round of assessment, for analysis of evidence of content validity, we obtained feedback from 23 judges, and the critical CVR considered was 0.39 (p=0.03). In the second round, there was feedback from 19 judges, and the critical CVR considered was 0.44 (p=0.04). In the third round, there was feedback from 17 judges, and the critical CVR considered was 0.52 (p=0.02)^(31)^.

For analysis of evidence of validity based on response processes, calculation was performed considering the 31 patients who remained in follow-up until the end of the study; therefore, the critical CVR considered was 0.35 (p=0.03)^(31)^.

## RESULTS

In the first stage of this study, based on the narrative review of literature, an article^(9)^ was used to support the construction of messages regarding barriers related to medication adherence and self-care, which were divided into the following domains: family support in treatment; complex therapeutic regimens; patients’ perception and knowledge of disease; medication costs; absence of symptoms; undesirable effects; treatment duration; required behavioral change in lifestyle habits; and access to healthcare services. Seven references were used to construct the content of the messages, including a guideline on physical activity^(32)^, cardiovascular prevention^(33)^ and cardioprotective diet^(34)^, in addition to four articles on medication adherence^(5,8,19,20)^.

Considering these barriers, 29 messages were developed, 27 of which were aimed at promoting adherence to treatment, a welcome message and reinforcement of the study’s objective, and another message that portrays a summarized approach to the main topics covered throughout follow-up.

Chart 1 shows the number of messages developed for each modifiable domain/barrier of medication adherence and self-care.

**Chart 1:** Domains, educational intervention and number of messages. São Paulo, SP, Brazil, 2020

Domain/adherence Barriers	Educational interventions	Number of messages created
Family support in treatment and complex therapeutic regimens	Approach to the support network to assist in the acquisition and organization of the use of medications.	2
Medication costs	Approach to the network for dispensing medications by the Brazilian Health System, whether of the basic or specialized component, as a guarantee of low-cost treatment.	1
Changes in habits	Approach to the need to change eating patterns, quit smoking and alcoholism as a complement to adherence to medication therapy.	1
Treatment duration	Generalist approach regarding the correct use of medications according to medical advice, the validity of prescriptions and provision of medications in a timely manner.	4
Patients’ perception and knowledge of disease	Specific approach regarding the use of medications after an acute coronary event, such as clopidogrel, acetylsalicylic acid and references to antihypertensives and statins.	7
Undesirable effects	Guidance regarding comorbidities and risk factors for CVD, such as hypertension, diabetes, sedentary lifestyle, smoking, with reinforcement of changes in lifestyle, diet, physical exercise and use of medications.	4
Absence of symptoms	Guidance on the need to follow treatment, with an alert to control the main risk factors for CVD (hypertension, dyslipidemia, smoking) which, sometimes, do not present symptoms.	3
Remembrance	Considerations regarding maintaining daily routines for regular use of medications, avoiding delays and failures.	5

**Figure 1:**
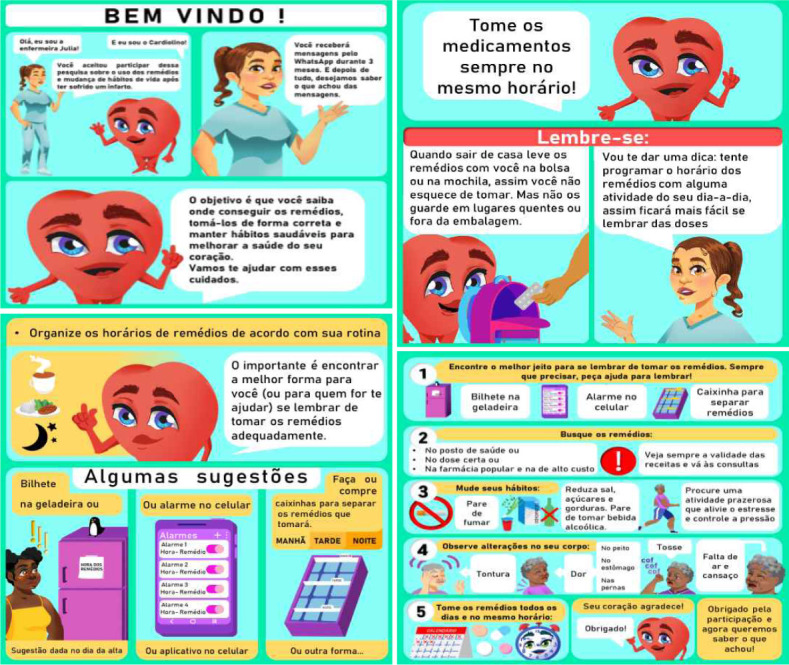
Examples of telephone messages developed to promote adherence and self-care in patients with coronary artery disease. São Paulo, SP, Brazil, 2020

To construct the messages, we sought to use respectful, yet colloquial and motivating language to address changes. Cartoons were used to construct the messages, creating characters that could convey the information, as shown in Figure 1, which exemplifies four message models developed and assessed by the judging committee to analyze evidence of content validity. The other messages can be viewed in Supplementary Material 1.

After development, the messages were sent to judges, with feedback from the participation of 23 professionals, including ten nurses specialized in cardiology, two pharmacists, five cardiologists, one social worker, one psychologist, one nutritionist, one nurse specialized in public health, one linguist and one physiotherapist, the majority of whom were female (60.9%), with specialist qualifications (39%), followed by doctoral (30%) and masters’ (17.4%) degrees, with 69% having up to ten years of experience in teaching, 86.9% in research, and 65.2% in care.

In the first round, a CVR value higher than 0.39 was achieved in all indicators and in all messages. However, given the pertinent suggestions made by judges regarding character characterization, timeless messages, care in relation to stereotype deconstruction, reinforcement of organizational methods, and reminders to take medications without standardizing times, respecting the individuality of life routines and the medical prescription for each patient, these were accepted and, by decision of the researchers, sent back to the committee of judges with modifications.

Furthermore, in the first round of message assessment, judges gave their opinions on the order in which the messages were sent and their suitability regarding their belonging to the domains/barriers to adherence previously classified by the researchers. Two messages previously categorized in the domain “patients’ perception and knowledge of disease” were allocated to the domain “change of habits”, according to judges’ suggestions.

In the second round, feedback was received from 19 other professionals, and CVR achieved was also higher than the critical value established for the number of judges. The suggestions at this stage were related to adjusting expressions, confirming services provided and emphasizing guidelines for changing habits. These suggestions were accepted by the researchers, and a new round of assessment was also decided upon.

In the third round, feedback was received from 17 judges, and all messages regarding the assessed indicators achieved a CVR above the established critical value. The suggestions focused on character appearance, letter color and size, and font standardization, with the final suggestions being accepted and the assessment by the committee completed.

In relation to clarity, practical relevance, theoretical relevance, suitability of language and figures with sentences, a final CVR of 0.99 was observed.

After obtaining evidence of content validity of the messages, the phase of analysis of evidence of validity based on response processes began. An active search for potential participants was carried out, which resulted in 535 patients hospitalized for different CVDs, of which 36 patients met the eligibility criteria and agreed to participate in the study. Table 2 shows the main sociodemographic characteristics of these participants.

**Table 2:** Sociodemographic characterization data of participants in the analysis phase of evidence of validity based on response processes. São Paulo, SP, Brazil, 2021

Variable	
Mean age (DO)	58.91 (7.6)
Male (%)	20 (55.6)
Education level (%)	
Incomplete elementary school	19 (52.8)
Complete elementary school	5 (13.9)
Complete high school	8 (22.2)
Higher education	4 (11.1)
Family income§ n (%)	
Less than 1 minimum wage	6 (16.7)
1 to 3 minimum wages	20 (55.6)
3 to 5 minimum wages	3 (8.3)
5 to 7 minimum wages	2 (5.6)
Refused to answer	5 (13.9)

*n = absolute frequency; % = relative frequency; SD = standard deviation; § = current minimum wage in reais (Brazilian currency) of R$1,100.00 in 2021, Brazil, 2021.*

Concerning access to healthcare services, it was identified that, for the most part, patients exclusively use public services, both for healthcare (88.9%) and for purchasing medications (50%).

Regarding clinical aspects, the majority were diagnosed with acute myocardial infarction with ST-segment elevation (44.4%), followed by acute myocardial infarction without ST-segment elevation (25%) and unstable angina (22.2%), and their main cardiovascular risk factors were sedentary lifestyle (77.8%), hypertension (77.7%), dyslipidemia (52.7%), diabetes mellitus (44.4%), active smoking (38.9%) and alcoholism (33.3%).

In relation to responsibility for the use of medications and family support relationships, 92% reported being responsible for organizing the maintenance of their treatment; of these, 36% also had help from other people (30% spouses and 17% children).

Of the 36 patients who agreed to participate in the study, five participants were lost, two due to being blocked from receiving messages, two unable to be contacted at the end of the study and one who reported not having read any messages due to lack of interest. In this regard, 31 participants assessed the messages and, therefore, the value of 0.35 (p=0.03) was considered as an acceptable critical CVR.

At this stage, some participants made important comments regarding sending messages, such as the need to use the zoom feature to read all messages, but they did not consider it a demotivating factor for reading, but rather a consequence of age and declining vision; attachment to the characters in the messages, as they are fun and relaxed figures; language considered easy to access and able to dialogue with different social classes objectively and clearly; the time of sending every three days was considered adequate, helping to create a routine, however, they sometimes considered it a little repetitive; the time of sending in the morning was considered quite adequate both for remembering and organizing routines. Moreover, there were comments regarding care representation, mentioning that, after discharge, they felt “abandoned” and had doubts, and due to the distance from scheduling the medical appointment, sending messages was the main source of information and connection with a healthcare professional, creating a relationship of comfort, security and support.

Table 3 shows CVR value after participants’ assessment in relation to the indicators assessed in this phase.

**Table 3:** Content Validity Ratio based on response processes according to participants’ answers (N=31). São Paulo, SP, Brazil, 2020

Indicator	CVR
Font size and type	0.87
Adequacy of figures with text	0.93
Sharpness of figures	1.00
Understanding of messages	0.87
Usefulness of messages	0.93
Adequacy/acceptability	0.74
Clarity of messages	0.87
Mean final CVR	0.88

*CVR – Content Validity Ratio.*

## DISCUSSION

Health education seeks ways to approach individuals in whom it is interested, considering that this individual is a participant in their own learning^(35)^. Currently, and accelerated by the pandemic context, the ways in which the population is approached and reached through virtual means, especially the use of cell phones, have forced healthcare professionals to adapt to this new educational context.

Data from 2023 on the Brazilian population’s access to internet service showed that 84% of Brazilians had access to the resource, the majority of whom were in urban areas^(36)^. There was also evidence of an increase in internet access by economic class C from 80% to 91% in 2023, and by economic classes D and E from 50% to 69%^(36)^. As for the justifications for not having access to the internet, the main barrier reported is still the costs involved, with 28% using this justification and another 20% reporting lack of knowledge about its use^(36,37)^.

Several studies have shown that medication adherence is improved when its barriers are overcome and, for this to occur, educational interventions must be carried out^(5,9,16,23,24,26)^, since instrumentalization of patients and family members regarding the importance of medication use, the ways to remember to use the medication, the places to acquire medications, and the incorporation of a healthy lifestyle can be improved with health education^(5,9,22,23)^.

The literature shows that one of the major predictors of failure in medication adherence is the difficulty in acquiring medications^(9)^, and to counter this barrier, some messages were developed to convey information about the free accessibility of medications, where to acquire them and places with lower economic values.

Other issues identified as hindering medication adherence include the duration of medication treatment, the time of medication appointment, and ways of remembering its use. An observational study assessed medication adherence in patients with acute coronary syndrome and its association with social support, and found that 88% of patients had a failure to remember their medications^(38)^. To portray this barrier in the messages, suggestions were listed that patients could include in their routine, such as using alarms on cell phones, using apps, or even charts posted in easily accessible places at home.

Other barriers addressed in the messages were related to the side effects of medications and the lack of easily identifiable symptoms, which can lead to treatment abandonment. A systematic review that included studies that analyzed people with diabetes mellitus showed that the presence of these barriers contributes to non-adherence^(39)^. Furthermore, the literature indicates that inadequate lifestyle habits, such as smoking, alcoholism and a sedentary lifestyle, can alter the level of medication adherence^(5-8)^.

The development and validity of health tools include the stages covered in this study^(27-30)^. It is known that the verification of evidence of content validity and based on response processes is one of the criteria used to analyze evidence of validity of an educational tool^(30)^. Assessment of tools by judges and the target audience is a complementary process to achieve quality results^(30,31)^. In this way, the validity process not only seeks to demonstrate whether the tool actually measures/promotes what it really proposes, but also allows for cultural adaptation and approximation of the instrument to be created/implemented to better reach the target audience^(27-30)^.

Therefore, obtaining agreement between judges and the target population regarding content, clarity, order of messages and delivery time is extremely important to ensure understanding of what is being presented and to achieve the proposed objective; in this case, adherence to drug treatment^(40)^.

The use of CVR proved to be sufficient and appropriate, especially because it is a quantitative data and provides a significance value in judges’ agreement, in addition to the cut-off point being dependent on the number of judges. Authors defend the use of CVR in relation to other methods of assessing agreement, such as the Content Validity Index, because it does not inflate judges’ agreement value^(30,31)^.

Another important aspect in the construction of health instruments is related to the adaptability and contextualization of the target audience, which must be guaranteed through the analysis phase of evidence of validity based on response processes^(30)^, which, in the present study, showed that the messages presented clear and easily accessible language, with eye-catching and fun figures, and that the cartoon format was a facilitator and motivator.

Recent studies have shown that the combination of images and shorter, simpler texts, such as cartoons, offers a good option for communicating about health issues^(41,42)^. Although pure text formats are commonly used to convey health information, a mix of text and images has been shown to be more effective, as information that is strictly composed of text can create a feeling of information overload in readers^(42)^. Researchers highlight that the use of narratives with humor, bright colors, characters representing the target audience and in harmony with local realities showed positive results in attracting and maintaining readers’ attention^(41-43)^. Therefore, information in cartoon format can support self-management actions in health, being accessible to different levels of education^(39,41-43)^.

When assessing acceptability, most patients reported that sending messages in the morning and every three days was satisfactory, which can be verified by the high CVR value. In the literature, no standard was found regarding the frequency of sending messages that is considered ideal, varying between daily messages and weekly or biweekly messages^(21,44)^. Therefore, there was also no consensus on the best follow-up time, with an average of three months^(17,22,25,28,44)^.

### Contributions to health, nursing, or public policy

Given the study data, the messages developed and validated may contribute to health education, especially for Brazilian Unified Health System users, as it emphasized how to access healthcare resources and supplies through the public network, and how such provision is available in the Greater São Paulo region. The popular channel itself, which is WhatsApp^®^, also aimed to strengthen the principles of information comprehensiveness and universality.

### Study limitations

The limitation of this study is related to the fact that the messages developed were based on the resources available in the Greater São Paulo region, requiring adaptation to other contexts and regions. Furthermore, the effect of sending these messages with regard to medication adherence was not assessed, which is considered a limiting factor and a future study perspective.

## CONCLUSIONS

After a narrative literature review, 29 telephone messages containing information for health education about CAD treatment were developed. The messages were developed based on potentially modifiable barriers to medication adherence, and three rounds of assessment by judges were necessary to reach the critical CVR. The messages were assessed by 31 patients hospitalized for CAD.

Further studies to increase the level of evidence of validity of the material developed must be carried out, mainly in relation to the assessment of the effect of this educational tool and the influence of factors on adherence to drug treatment.

## Data Availability

The research data are available upon request, and most of the data are presented within the body of the article.
