# Supplementary material for: Telephone messages for medication adherence and self-care for individuals with coronary heart disease
Source: Rev Bras Enferm. 2025 Nov 3;78(4):e20240351. doi: 10.1590/0034-7167-2024-0351 (PMC12584950; doi:10.1590/0034-7167-2024-0351)
Supplement: Translation

# Translation: Mensagens telefônicas para adesão medicamentosa e autocuidado para indivíduos com doença coronariana

**DOI:** 10.1590/0034-7167-2024-0351pt

**Authors:** Luize Maximo e Melo, Vinícius Batista Santos, Juliana de Lima Lopes

**Affiliations:** I Universidade de São Paulo. São Paulo, São Paulo, Brasil; II Universidade Federal de São Paulo. São Paulo, São Paulo, Brasil

**Keywords:** Doença das Coronárias, Adesão à Medicação, Educação em Saúde, Telefone Celular, Estudos de Validação

## Abstract

**Objetivo:**

desenvolver e analisar as evidências de validade de mensagens telefônicas para promoção da adesão medicamentosa e do autocuidado de pacientes com doença arterial coronariana.

**Método:**

estudo metodológico, realizado em três etapas: I) revisão de literatura e desenvolvimento das mensagens; II) avaliação das evidências de validade de conteúdo por juízes; III) avaliação das evidências de validade baseada nos processos de resposta por pacientes submetidos à intervenção coronariana percutânea. Foi calculada a Razão de Validade de Conteúdo para análise das evidências de validade.

**Resultados:**

: foram desenvolvidas 29 mensagens telefônicas relacionadas às barreiras de adesão medicamentosa e de autocuidado, que foram avaliadas por 23 juízes e 31 pacientes, observando-se valores adequados da Razão de Validade de Conteúdo.

**Conclusão:**

as mensagens foram desenvolvidas e alcançaram adequadas evidências de validade, e poderão ser uma ferramenta educativa para essa população.

## INTRODUÇÃO

As doenças cardiovasculares (DCVs) são as principais causas de morte no Brasil e no mundo. Entre elas, a doença arterial coronariana (DAC) é responsável por elevada taxa de hospitalização^(1,2)^. Para o controle e redução das taxas de morbimortalidade por DAC, são necessárias a implementação de medidas preventivas que estimulem mudanças no estilo de vida e a adesão do paciente ao regime terapêutico, uma vez que a não adesão ao tratamento está associada a piores desfechos^(3)^.

Historicamente, a adesão ao tratamento é um conceito complexo que migrou de abordagens autoritárias até a aceitação de autogestão do tratamento pelo indivíduo^(4-8)^. A adesão é, então, definida como o comportamento que coincide com o plano de cuidados acordado entre os profissionais de saúde e o paciente^(5-8)^, sendo que a adesão farmacológica é definida pela utilização de pelo menos 80% dos medicamentos prescritos, observando horário, dose e tempo de tratamento^(5-8)^.

A não adesão ao tratamento é uma questão de grande complexidade, estando relacionada a aspectos de diferentes naturezas, ou seja, ligados ao tratamento em si (complexidade da farmacoterapia, duração do tratamento e intensidade das reações adversas), à condição de saúde (doenças crônicas e condições assintomáticas), ao paciente (limitações cognitivas e funcionais, conhecimento sobre as condições de saúde e sobre os medicamentos, dificuldades físicas e motoras), a fatores sociais e econômicos (falta de suporte familiar e social, crenças culturais, falta de acesso aos serviços de saúde, falta de acesso aos medicamentos), e a fatores relacionados ao sistema e ao vínculo com a equipe de saúde^(9)^.

Analisar o comportamento do uso dos medicamentos prescritos exige amplas abordagens que contemplem a importância do autocuidado, da autogestão e do automonitoramento em saúde em saúde^(4,7,8)^. Por isso, esta pesquisa utilizou como referencial teórico a teoria de média abrangência de Dorothéa Orem, que considera que o autocuidado inclui ações iniciadas e executadas pelos indivíduos em seu próprio benefício para manutenção da vida e do bem-estar, contribuindo com o propósito de integridade das funções e de desenvolvimento humano^(10-13)^. Essa teoria descreve que os indivíduos desejam e podem se tornar aptos ao seu autocuidado, podendo os profissionais de enfermagem assumir esta tarefa quando não estão em condições de fazê-lo^(12)^ e atuar por meio da educação em saúde, possibilitando a aderência às condutas preventivas e terapêuticas (autogestão em saúde), e ao monitoramento de sua saúde (automonitoramento), tornando-o agente ativo de seu próprio cuidado e facilitando, dessa maneira, o autocuidado^(10-13)^.

Com o incremento da tecnologia e da popularização dos recursos de mensagem por telefonia móvel (*WhatsApp* ^®^) e das regulamentações legais^(14-17)^, tem-se o acompanhamento em saúde por serviços de telefonia móvel, com a finalidade de promover maior acessibilidade aos usuários dos serviços de saúde, por fornecer uma via de comunicação de baixo custo de maneira aberta e imediata, expandir o acesso aos cuidados de saúde, reduzir os custos e as barreiras geográficas entre o paciente e o profissional de saúde, e superar as dificuldades com o tratamento^(16-18)^.

Na literatura, encontram-se estudos primários e revisões sistemáticas de literatura que objetivaram avaliar o efeito das mensagens por celular, sendo identificado que essas intervenções propiciam o aumento nas mudanças de comportamentos em relação ao estilo de vida (autocuidado em saúde), aumento da adesão medicamentosa (autogestão em saúde) e melhor monitoramento dos sinais clínicos (automonitoramento em saúde)^(19-25)^. Em revisão recente realizada pela Cochrane (2024)^(26)^ sobre o uso de mensagens telefônicas em educação em saúde para coronariopatas, evidenciou-se importante lacuna de estudos que demonstrassem o efeito desta intervenção, principalmente em populações de baixa renda, e também a falta de estudos que avaliassem as evidências de validade desses materiais educacionais visando ao alcance de resultados positivos em saúde^(26)^.

Diante do fato de que a inserção das tecnologias da informação e comunicação em contextos educacionais em saúde pode ser um facilitador para o autogerenciamento, automonitoramento e autocuidado em saúde, faz-se necessário que essas tecnologias sejam submetidas a técnicas psicométricas para que se avaliem as evidências de validade desses materiais^(27,28)^. Sendo assim, no melhor do conhecimento dos autores, não foi identificado nenhum estudo brasileiro que desenvolveu e analisou as evidências de validade de mensagens telefônicas visando à adesão medicamentosa para pacientes com doença coronariana com foco nas principais barreiras relacionadas à adesão.

Considera-se o caráter de pioneirismo no estudo da enfermagem brasileira, tanto por vincular uma ferramenta tecnológica digital quanto por seu aperfeiçoamento e sistematização metodológica, por meio da análise das evidências de validade tanto por especialistas quanto pela população-alvo.

## OBJETIVO

Desenvolver e analisar as evidências de validade de mensagens telefônicas para promoção da adesão medicamentosa e do autocuidado de pacientes com DAC.

## MÉTODOS

### Aspectos éticos

Esta pesquisa foi conduzida de acordo com as diretrizes de ética nacionais e internacionais, sendo aprovada pelo Comitê de Ética em Pesquisa com Seres Humanos da Universidade Federal de São Paulo. Foi conduzida de forma ética, sigilosa e somente com autorização formal de cada participante da pesquisa, por meio da assinatura do Termo de Consentimento Livre e Esclarecido (TCLE), obtido por meio escrito e eletrônico.

### Desenho do estudo

Trata-se de estudo metodológico cuja estratégia sistemática utiliza o conhecimento existente de um fenômeno para elaborar, adaptar, desenvolver, melhorar e avaliar um instrumento quanto à sua confiabilidade, validade, utilidade e replicabilidade^(27,29,30)^. A literatura destaca algumas etapas para a elaboração de instrumentos/ferramentas educacionais que partem da construção de evidências de validade de conteúdo (estabelecimento da estrutura conceitual/variável latente, definição dos objetivos, construção dos itens/tópicos da ferramenta, seleção e organização dos itens/tópicos, estruturação do instrumento/ferramenta, e avaliação por comitê de juízes heterogêneo), seguidas de envolvimento da população-alvo para determinação das evidências de validade baseadas no processo de resposta^(27-30)^.

Este estudo se desenrolou ao longo de maio de 2020 até janeiro de 2022. Os dados foram coletados com pacientes internados nas Unidades de Cardiologia Clínica de um hospital-escola do serviço público de saúde de nível terciário da cidade de São Paulo.

### População e amostra

Para o processo de análise das evidências de validade de conteúdo, foi selecionado um comitê de juízes com titulação mínima de especialista e experiência de mais de quatro anos de atuação na área específica de cardiologia ou de saúde coletiva^(27,29,30)^. O recrutamento foi feito pelo tipo bola de neve, com um primeiro estrato por conhecimento dos autores desta pesquisa e pela seleção na Plataforma *Lattes*, a fim de agregar um contingente heterogêneo de especialistas, ocorrendo entre maio e novembro de 2020.

Na fase de avaliação das evidências de validade baseada nos processos de resposta, foram selecionados e acompanhados pacientes entre o período de março de 2021 e janeiro de 2022. Os critérios de inclusão foram pacientes hospitalizados por alguma manifestação da DAC, com idade superior ou igual a 18 anos, que foram submetidos à intervenção coronária percutânea pela primeira vez, usuários do sistema de envio de mensagens por telefone móvel (*WhatsApp* ^®^) e ser capaz de falar e entender a língua portuguesa. Não foram considerados para essa etapa os pacientes que apresentavam declínio cognitivo grave e/ou afecções psiquiátricas prévias relatadas no prontuário. Foram considerados como perda de seguimento aqueles em que se detectou falha de recebimento das mensagens ou solicitação para saída da pesquisa, falha em contato telefônico para aplicação dos questionários e entrevista ao final, e readmissão por novo evento isquêmico e óbito.

### Protocolo de estudo

As etapas desenvolvidas nesta pesquisa foram: 1) revisão narrativa de literatura e desenvolvimento das mensagens para o envio para telefones móveis; 2) avaliação das evidências de validade de conteúdo por um comitê de juízes especialistas na área da cardiologia; 3) avaliação das evidências de validade baseada nos processos de resposta pelos pacientes submetidos à intervenção coronária percutânea.

Entende-se por avaliação das evidências de validade de conteúdo a relação entre o conteúdo do material/instrumento desenvolvido com o fenômeno de interesse, ou seja, é uma verificação se o material/instrumento se destina ao construto principal em relação à relevância teórica, pertinência prática e clareza, além da avaliação da aparência geral e até recursos visuais utilizados na elaboração^(29,30)^. Já a análise das evidências de validade baseada nos processos de resposta está relacionada à percepção do respondente em relação ao material desenvolvido, e pode ser realizada, entre outras técnicas, por meio de entrevista com a população-alvo. Essa análise é bastante importante, por ser o primeiro momento de contato da ferramenta com o público a qual se destina, buscando um equilíbrio entre a perspectiva dos juízes e do grupo populacional. Para isso, baseia-se em diversos tipos de indicadores, tais como compreensibilidade, abrangência do material, organização geral e relevância^(30)^.

#### Primeira etapa

A primeira etapa deste estudo contou com revisão narrativa de literatura e elaboração das mensagens. A revisão foi realizada nas bases de dados PubMed, SciELO e Biblioteca Virtual em Saúde, utilizando-se os descritores de assunto “Doença das Coronárias” e “Adesão à Medicação” e “Educação em Saúde”. Foram selecionados artigos sobre a temática desenvolvidos nos últimos cinco anos e depositados em tais bases de dados. A busca e a seleção dos artigos foram identificadas pela triagem de títulos e resumos realizados por um dos autores do estudo, que extraiu as intervenções educacionais visando à solução das barreiras de adesão potencialmente modificáveis e promoção do autocuidado na população de pacientes com DAC.

Após a identificação das intervenções educacionais, os autores desenvolveram as mensagens telefônicas contendo textos e figuras. Posteriormente, as figuras foram finalizadas e desenhadas por um ilustrador profissional.

#### Segunda etapa

Os juízes foram convidados por correio eletrônico, sendo explicados os objetivos da pesquisa. Após o aceite, foi enviado um *link* do *Google Forms* ^®^ por e-mail, no qual havia o TCLE eletrônico, o questionário de caracterização sociodemográfica e profissional (idade, sexo, nível acadêmico e tempo de experiência na assistência, no ensino e ou na pesquisa), seguido do instrumento com as mensagens desenvolvidas.

Para análise das mensagens, uma escala do tipo Likert de quatro pontos foi utilizada, sendo o escore 1 não adequado, 2, moderadamente adequado, 3, adequado, e, 4 totalmente adequado. Os juízes avaliaram cada mensagem em relação à relevância teórica das informações contidas nas mensagens, clareza das informações, pertinência prática, adequação das figuras com o texto das mensagens, tamanho da fonte e nitidez^(27,28)^. Ao final, foi deixado um campo para sugestões de mudanças e justificativas da pontuação dada.

#### Terceira etapa

A inclusão dos pacientes foi realizada no dia anterior a alta hospitalar, sendo, nesse contato, explicados os objetivos da pesquisa, lido, entregue e assinado o TCLE, e no caso de aceite, aplicou-se o primeiro instrumento contendo dados de caracterização sociodemográfica e clínica por meio de entrevista estruturada. Este instrumento continha dados relacionados ao sexo, idade, grau de formação, estado civil, peso e altura, antecedentes pessoais, hábitos de vida, acesso ao serviço de saúde, e dados referentes ao uso de medicamentos (responsabilização do uso das medicações e relações de apoio familiar). Em seguida, foi solicitada a identificação do número de telefone móvel para uso do recurso *WhatsApp* ^®^. Logo ao ser adicionado ao estudo, o paciente recebia uma primeira mensagem confirmatória do propósito da pesquisa para verificação de seu envio correto.

O envio das mensagens educativas era iniciado no dia seguinte da alta dos participantes, sendo mantidas por um prazo de 94 dias, sendo que, na primeira semana após a alta, as mensagens eram enviadas diariamente; em seguida, foram espaçadas com intervalo de três dias. Foi utilizado também o recurso de “mensagens-coringa”, que eram conteúdos de reforço e/ou motivação quanto às mudanças de comportamento e que se repetiam ao longo do estudo de forma intercalada. O tempo de 94 dias foi estabelecido após a etapa de validação de evidências de conteúdo ser concluída e, então, estar pronto o número total de mensagens que, intercaladas pelas mensagens-coringa e enviadas com o intervalo pré-definido, totalizaram 94 dias de acompanhamento.

Após esse período, foi realizado um contato telefônico para uma entrevista de avaliação das mensagens em relação ao tamanho e tipo de fonte, adequação das figuras com o texto, nitidez das imagens e entendimento, utilidade, e adequação e clareza dessas mensagens. Para tanto, aplicou-se um instrumento elaborado pelos autores contendo uma escala do tipo Likert de 4 pontos, sendo o escore 1 não adequado, 2, moderadamente adequado, 3, adequado, e 4, totalmente adequado. Juntamente, fez-se análise qualitativa das respostas da entrevista por meio de uma pergunta aberta quanto à usabilidade do recurso.

### Análise dos resultados e estatística

Os dados coletados foram tratados em uma planilha do programa *Microsoft Excel* ^®^ e analisados pelos pesquisadores utilizando o programa estatístico *Statistical Package for the Social Sciences* ^®^, versão 22.0. Foi realizada a estatística descritiva das variáveis apresentadas, sendo que as variáveis categóricas foram apresentadas em frequências absolutas e relativas, e as variáveis numéricas, em média e desvio padrão ou mediana e valores mínimos-máximos, de acordo com a distribuição da amostra pelo teste de normalidade de Shapiro-Wilk.

Para análise das evidências de validade de conteúdo nos processos de resposta, foi calculada a Razão de Validade de Conteúdo (CVR - *Content Validity Ratio*) para cada indicador avaliado de mensagem, conforme fórmula CVR= Ne- (N/2)/N/2, sendo Ne o número de juízes e pacientes que assinalaram o escore 3 e 4, e N, o número de respondentes^(31)^.

O valor crítico aceitável foi dependente do número de juízes respondentes, conforme recomendado por Ayre em 2014, sendo recomendado um número mínimo de cinco juízes^(31)^. Na primeira rodada de avaliação, para análise das evidências de validade de conteúdo, obtivemos uma devolutiva de 23 juízes, e o CVR crítico considerado foi de 0,39 (p=0,03). Na segunda rodada, houve a devolutiva de 19 juízes, e o CVR crítico considerado foi de 0,44 (p=0,04). Na terceira rodada, houve a devolutiva de 17 juízes, e o CVR crítico considerado foi de 0,52 (p=0,02)^(31)^.

Para análise das evidências de validade baseada nos processos de resposta, o cálculo foi realizado considerando os 31 pacientes que mantiveram o seguimento até o final do estudo; portanto, o CVR crítico considerado foi de 0,35 (p=0,03)^(31)^.

## RESULTADOS

Na primeira etapa deste estudo, baseando-se na revisão narrativa de literatura realizada, foi utilizado um artigo^(9)^ que sustentou a construção das mensagens em relação às barreiras relacionadas à adesão medicamentosa e ao autocuidado, tendo sido divididas nos seguintes domínios: auxílio da família no tratamento; esquemas terapêuticos complexos; percepção e conhecimento da doença pelo paciente; custos dos medicamentos; ausência de sintomas; efeitos indesejáveis; duração do tratamento; mudança comportamental requerida nos hábitos de vida; e acesso ao serviço de saúde. Para construção do conteúdo das mensagens, foram utilizados sete referências, sendo um *guideline* sobre atividade física^(32)^, prevenção cardiovascular^(33)^ e alimentação cardioprotetora^(34)^, além de quatro artigos sobre adesão medicamentosa^(5,8,19,20)^.

Diante dessas barreiras, foram desenvolvidas 29 mensagens, sendo 27 direcionadas à promoção da adesão ao tratamento, uma mensagem de boas-vindas e reforço do objetivo do estudo, e outra mensagem que retrata uma abordagem resumida dos principais assuntos contemplados ao longo do acompanhamento.

No Quadro 1, encontra-se o número de mensagens desenvolvidas para cada domínio/barreira modificável de adesão medicamentosa e autocuidado.

**Quadro 1:** Domínios, intervenção educativa e número de mensagens. São Paulo, SP, Brasil, 2020

Domínio/barreiras de adesão	Intervenções educativas	Número de mensagens elaboradas
Auxílio da família no tratamento e esquemas terapêuticos complexos	Abordagem sobre a rede de apoio para auxílio na aquisição e organização do uso dos medicamentos.	2
Custos dos medicamentos	Abordagem sobre a rede de dispensação de remédios pelo Sistema Único de Saúde, seja do componente básico ou do especializado como garantia do tratamento a baixo custo.	1
Mudanças de hábitos	Abordagem sobre necessidade de mudança no padrão alimentar, cessação do tabagismo e do etilismo como complementares a adesão da terapia medicamentosa.	1
Duração do tratamento	Abordagem generalista quanto ao seguimento correto do uso das medicações conforme orientação médica, à validade das receitas e à providência das medicações em tempo hábil. Abordagem específica quanto ao uso das medicações após evento agudo coronariano, a exemplo do clopidogrel, ácido acetilsalicilico e de referências a anti-hipertensivos e estatinas.	4
Percepção e conhecimento da doença pelo paciente	Orientações quanto às comorbidades e fatores de risco para doença cardiovascular, como hipertensão arterial sistêmica, diabetes, sedentarismo, tabagismo, com reforço a mudanças de estilo de vida, alimentação, prática de exercícios físicos e uso das medicações.	7
Efeitos indesejáveis	Orientação quanto à identificação de efeitos indesejáveis para o não abandono do tratamento, referindo alguns sinais mais frequentes, como dispepsia, tosse seca, tontura e mialgia.	4
Ausência de sintomas	Orientação quanto à necessidade de seguimento do tratamento, com alerta para o controle dos principais fatores de risco para doença cardiovascular (hipertensão arterial sistêmica, dislipidemia, tabagismo) que, por vezes, não apresentam sintomas.	3
Recordação	Considerações quanto à manutenção de rotinas diárias para uso regular das medicações, evitando atrasos e falhas.	5

Para construção das mensagens, buscou-se uma linguagem respeitosa, porém coloquial e motivadora, para o enfrentamento de mudanças. Foi utilizado o recurso de *charge* para a construção das mensagens, criando-se personagens que pudessem transmitir as informações, conforme Figura 1, que exemplifica quatro modelos de mensagens desenvolvidas e avaliadas pelo comitê de juízes para análise das evidências de validade de conteúdo. As demais mensagens podem ser visualizadas no Material Suplementar 1.

**Figura 1:** Exemplos das mensagens telefônicas desenvolvidas para promoção de adesão e autocuidado nos pacientes com doença arterial coronariana. São Paulo, SP, Brasil, 2020

Após o desenvolvimento, as mensagens foram enviadas aos juízes, com devolutiva de participação de 23 profissionais, sendo dez enfermeiros especialistas em cardiologia, dois farmacêuticos, cinco médicos cardiologistas, um assistente social, uma psicóloga, uma nutricionista, um enfermeiro especialista em saúde coletiva, um linguista e um fisioterapeuta, sendo a maioria do sexo feminino (60,9%), com titulação de especialista (39%), seguida de doutores (30%) e mestres (17,4%), sendo que 69% tinham até dez anos de experiência no ensino, 86,9%, na pesquisa, e 65,2%, na assistência.

Na primeira rodada, alcançou-se um valor de CVR superior a 0,39 em todos os indicadores e em todas as mensagens, entretanto, diante das pertinentes sugestões realizadas pelos juízes em relação à caracterização dos personagens, mensagens atemporais, cuidados em relação à desconstrução de estereótipos, reforço a modos de organização, e recordação para tomada de medicações sem padronização de horários, respeitando a individualidade das rotinas de vida e a prescrição médica para cada paciente, essas foram acatadas e, por decisão dos pesquisadores, reenvidas ao comitê de juízes com as modificações.

Ainda, na primeira rodada de avaliação das mensagens, os juízes opinaram quanto à ordem de envio das mensagens e à adequação quanto ao pertencimento dessas aos domínios/barreiras de adesão previamente classificados pelos pesquisadores. Duas mensagens categorizadas anteriormente no domínio “percepção e conhecimento da doença pelo paciente” foram alocadas no domínio “mudança de hábitos”, de acordo com a sugestão dos juízes.

Na segunda rodada, houve a devolutiva de outros 19 profissionais, e o CVR alcançado também foi superior ao valor crítico estabelecido para o número de juízes. As sugestões nessa etapa foram em relação ao ajuste de expressões, confirmação de serviços disponibilizados e ênfase nas orientações de mudança de hábitos. Essas sugestões foram acatadas pelos pesquisadores, sendo optada também por uma nova rodada de avaliação.

Na terceira rodada, foram recebidas as devolutivas de 17 juízes, e todas as mensagens em relação aos indicadores avaliados alcançaram um CVR acima do valor crítico estabelecido. As sugestões focaram na aparência dos personagens, cores e tamanhos de letras, e padronização de fontes, tendo sido acatadas as sugestões finais e finalizada a avaliação pelo comitê.

Em relação à clareza, pertinência prática, relevância teórica, adequação da linguagem e das figuras com as frases, observou-se um CVR final de 0,99.

Após o alcance das evidências de validade de conteúdo das mensagens, iniciou-se a fase da análise das evidências de validade baseada nos processos de resposta. Foi feita a busca ativa de possíveis participantes, que resultou em 535 pacientes internados por diferentes DCVs, sendo que 36 pacientes cumpriam os critérios de elegibilidade e aceitaram participar do estudo. A Tabela 2 evidencia as principais características sociodemográficas desses participantes.

**Tabela 2:** Dados de caracterização sociodemográfica dos participantes na fase de análise das evidências de validade baseada nos processos de resposta. São Paulo, SP, Brasil, 2021

Variável	
Idade média (DP)	58,91 (7,6)
Sexo masculino n (%)	20 (55,6)
Escolaridade n (%)	
Ensino fundamental incompleto	19 (52,8)
Ensino fundamental completo	5 (13,9)
Ensino médio completo	8 (22,2)
Ensino superior	4 (11,1)
Renda familiar§ n (%)	
Menos de 1 salário mínimo	6 (16,7)
1 a 3 salários mínimos	20 (55,6)
3 a 5 salários mínimos	3 (8,3)
5 a 7 salários mínimos	2 (5,6)
Recusou responder	5 (13,9)

*n = frequência absoluta; % = frequência relativa; DP = desvio padrão; § = salário mínimo vigente em reais de R$1.100,00 no ano de 2021, Brasil, 2021.*

No que tange ao acesso a serviços de saúde, foi identificado que, majoritariamente, os pacientes recorrem exclusivamente aos serviços públicos, tanto para atendimentos de saúde (88,9%) quanto para aquisição dos medicamentos (50%).

No que diz respeito aos aspectos clínicos, a maioria foi diagnosticada com infarto agudo do miocárdio com supradesnivelamento do segmento ST (44,4%), seguido de infarto agudo do miocárdio sem supradesnivelamento do segmento ST (25%) e angina instável (22,2%), e tinham como principais fatores de risco cardiovascular o sedentarismo (77,8%), hipertensão arterial sistêmica (77,7%), dislipidemia (52,7%), diabetes mellitus (44,4%), tabagismo ativo (38,9%) e etilismo (33,3%).

Em relação à responsabilização do uso das medicações e relações de apoio familiar, 92% referiram serem responsáveis pela organização para manutenção do seu tratamento; desses, 36% contavam também com ajuda de outras pessoas (30% cônjuges e 17% filhos).

Dos 36 pacientes que aceitaram participar do estudo, houve perda de cinco participantes, sendo dois por bloqueio de recebimento de mensagens, dois sem possibilidade de contato ao final do estudo e um por relato de não ter lido nenhuma mensagem por desinteresse. Nesse sentido, 31 participantes avaliaram as mensagens e, dessa forma, considerou-se o valor de 0,35 (p=0,03) como CVR crítico aceitável.

Nesta fase, alguns participantes realizaram comentários importantes em relação ao envio das mensagens, como a necessidade de usar o recurso de zoom para ler todas as mensagens, porém não consideraram um fator desmotivador para leitura, mas sim uma consequência da idade e do declínio da visão; apego pelas personagens das mensagens, por serem figuras divertidas e descontraídas; linguagem considerada de fácil acesso e que pode dialogar com diferentes classes sociais de forma objetiva e clara; o tempo de envio a cada três dias foi considerado adequado, auxiliando na criação de rotina, entretanto, por vezes, consideraram pouco repetitivas; o horário de envio pela manhã foi considerado bastante adequado tanto para recordação quanto para organização de rotinas. Além disso, houve comentários quanto à representação de cuidado, sendo referido que, após a alta, sentiram-se “abandonados” e com dúvidas, e devido à distância do agendamento da consulta médica, o envio das mensagens foi a principal fonte de informação e vínculo com um profissional de saúde, criando uma relação de conforto, segurança e suporte.

Na Tabela 3, é demonstrado o valor do CVR após a avaliação dos participantes em relação aos indicadores avaliados nesta fase.

**Tabela 3:** Razão de Validade de Conteúdo baseada nos processos de respostas conforme respostas dos participantes (N=31). São Paulo, SP, Brasil, 2020

Indicador	CVR
Tamanho e tipo de fonte	0,87
Adequação das figuras com o texto	0,93
Nitidez das figuras	1,00
Entendimento das mensagens	0,87
Utilidade das mensagens	0,93
Adequação/aceitabilidade	0,74
Clareza das mensagens	0,87
CVR médio final	0,88

*CVR – Content Validity Ratio.*

## DISCUSSÃO

A educação em saúde busca caminhos de aproximação com o indivíduo ao qual se tem interesse, considerando que esse indivíduo seja um participante do seu próprio aprender^(35)^. Na atualidade, e acelerados pelo contexto de pandemia, os modos de aproximação e alcance da população para os meios virtuais, em especial o uso do celular, forçaram uma necessidade de adaptação dos profissionais de saúde a esse novo contexto educativo.

Dados de 2023 sobre o acesso da população brasileira ao serviço de internet mostraram que 84% dos brasileiros tinham acesso ao recurso, sendo em sua maioria na zona urbana^(36)^. Foi evidenciado também um aumento do acesso à internet pela classe econômica C de 80% para 91% em 2023, e pelas classes econômicas D e E de 50% para 69%^(36)^. Quanto às justificativas sobre o não acesso à internet, a principal barreira relatada ainda são os custos envolvidos, sendo que 28% utilizaram essa justificativa e outros 20% referiram desconhecimento em seu uso^(36,37)^.

Diversos estudos demonstraram que a adesão medicamentosa é melhorada quando suas barreiras são superadas e, para que isto ocorra, intervenções educativas devem ser realizadas^(5,9,16,23,24,26)^, uma vez que a instrumentalização dos pacientes e familiares quanto à importância do uso da medicação, as formas de recordação para o uso da medicação, os locais para aquisição dos medicamentos, e a incorporação de um estilo de vida saudável podem ser melhorados com a educação em saúde^(5,9,22,23)^.

A literatura evidencia que um dos grandes preditores de falha na adesão medicamentosa é a dificuldade na aquisição dos medicamentos^(9)^, e para contrapor essa barreira, algumas mensagens foram desenvolvidas para transmitir informações sobre a acessibilidade gratuita dos medicamentos, local para aquisição dos mesmos e locais com menores valores econômicos.

Outros assuntos apontados como dificultadores para adesão medicamentosa são tempo de tratamento medicamentoso, horário de aprazamento e formas de recordação para seu uso. Estudo observacional avaliou adesão medicamentosa em pacientes com síndrome coronariana aguda e sua associação com o suporte social, e identificou que 88% dos pacientes apresentavam falha na recordação dos medicamentos^(38)^ e, para retratar essa barreira nas mensagens, foram elencadas sugestões que o paciente poderia incluir em sua rotina, como o uso de alarme nos celulares, uso de aplicativos ou mesmo tabelas fixadas em locais de fácil acesso no domicílio.

Outras barreiras abordadas nas mensagens foram em relação aos efeitos colaterais dos medicamentos e ao não aparecimento de sintomas facilmente identificáveis, o que pode ocasionar o abandono do tratamento. Revisão sistemática que incluiu estudos que analisaram pessoas com diabetes mellitus mostrou que a presença dessas barreiras contribui para a não adesão^(39)^. Além disso, a literatura aponta que hábitos de vida inadequados, como o tabagismo, alcoolismo e sedentarismo, podem alterar o nível de adesão medicamentosa^(5-8)^.

A elaboração e validação de ferramentas na área da saúde contam com as etapas perpassadas neste estudo^(27-30)^. É sabido que a verificação de evidências de validade de conteúdo e baseadas nos processos de resposta é um dos critérios utilizados para análise das evidências de validade de uma ferramenta educacional^(30)^. A avaliação de ferramentas por juízes e pelo público-alvo é processo complementar para se atingir resultados de qualidade^(30,31)^. Dessa forma, o processo de validação não somente busca evidenciar se a ferramenta realmente mede/promove o que realmente se propõe, mas também permite adequação e aproximação cultural do instrumento a ser criado/implementado para melhor alcance ao público-alvo^(27-30)^.

Logo, obter concordância entre juízes e a população-alvo com relação ao conteúdo, clareza, ordem das mensagens e tempo de envio é extremamente importante para garantir o entendimento do que está sendo apresentado e para se alcançar o objetivo proposto; neste caso, a adesão ao tratamento medicamentoso^(40)^.

O uso do CVR se mostrou suficiente e adequado, até mesmo por ser um dado quantitativo e por trazer um valor de significância na concordância dos juízes, além do ponto de corte ser dependente do número de juízes. Autores defendem a utilização do CVR em relação a outros métodos de avaliação de concordância, como o Índice de Validade de Conteúdo, por não inflacionar o valor de concordância dos juízes^(30,31)^.

Outro aspecto importante na construção de instrumentos em saúde está relacionado à adaptabilidade e contextualização do público-alvo, que deve ser garantida por meio da fase de análise das evidências de validade baseada nos processos de resposta^(30)^, que, no presente estudo, evidenciou que as mensagens apresentavam uma linguagem clara e de fácil acesso, com figuras chamativas e divertidas, sendo que o formato de *charge* foi um facilitador e motivador.

Estudos recentes têm demonstrado que a mistura de imagens e textos mais simplificados e curtos, como as *charges*, oferece uma boa opção de comunicação sobre temas de saúde^(41,42)^. Embora os formatos de textos puros sejam comumente utilizados para transmitir informações de saúde, um misto de textos e imagens tem demonstrado ser mais eficiente, uma vez que informações estritamente compostas por textos podem gerar uma sensação de sobrecarga de informação aos leitores^(42)^. Pesquisadores destacam que o emprego de narrativas com humor, cores chamativas, personagens de representação do público-alvo e em harmonia com as realidades locais mostrou resultados positivos na atração e manutenção da atenção dos leitores^(41-43)^. Logo, uma informação em formato de *charge* pode apoiar ações de autogerenciamento em saúde, sendo acessíveis para diferentes níveis de escolaridade^(39,41-43)^.

Ao avaliar a aceitabilidade, a maioria dos pacientes relatou que o envio das mensagens no período da manhã e a cada três dias foi satisfatório, o que pode ser verificado pelo alto valor do CVR. Na literatura, não foi encontrado um padrão em relação à frequência de envio das mensagens que seja considerado ideal, variando entre mensagens diárias e mensagens semanais ou quinzenais^(21,44)^. Assim, também não houve consenso de melhor tempo de acompanhamento, tendo uma média de três meses^(17,22,25,28,44)^.

### Contribuições para as áreas da saúde, enfermagem, ou políticas públicas

Diante dos dados do estudo, as mensagens desenvolvidas e validadas poderão contribuir para a educação em saúde, principalmente para usuários do Sistema Único de Saúde, pois foi enfatizado como acessar os recursos assistenciais e de insumos pela rede pública, e como tal oferta se dispõe na região da Grande São Paulo. O próprio canal de popularidade, que é o *WhatsApp* ^®^, também visou fortalecer os princípios de integralidade e universalidade da informação.

### Limitações do estudo

A limitação deste estudo está relacionada ao fato de que as mensagens elaboradas foram baseadas nos recursos disponíveis na região da Grande São Paulo, necessitando de adaptação para outros contextos e regiões, além de que não foi avaliado o efeito do envio dessas mensagens no que tange à adesão medicamentosa, sendo considerado um fator limitante e uma perspectiva futura de estudo.

## CONCLUSÕES

Foram elaboradas, após revisão narrativa de literatura, 29 mensagens telefônicas contendo informações para educação em saúde sobre o tratamento de DAC. As mensagens foram desenvolvidas a partir das barreiras potencialmente modificáveis para adesão medicamentosa, e foram necessárias três rodadas de avaliação pelos juízes para alcance do CVR crítico. As mensagens foram avaliadas por 31 pacientes hospitalizados por DAC.

Estudos posteriores para aumentar o nível de evidências de validade do material desenvolvido devem ser realizados, principalmente em relação à avaliação do efeito dessa ferramenta educacional e à influência de fatores na adesão ao tratamento medicamentoso.

## Data Availability

Os dados de pesquisa estão disponíveis mediante solicitação e a maior parte dos dados estão disponíveis no corpo do artigo.
